# Forecasting Sex

**Published:** 1898-07

**Authors:** 


### FORECASTING SEX.

Professor Schenck, either through his misfortune or fault, has much to answer for. By the publication to the world of his theory that he is able to produce at will a child of either sex without giving proof of the efficacy of his methods, he has incited other members of the medical profession, induced in it, is to be feared, by the prospect of self-advertisement and by the hope of gain, to propound propositions of a like nature. To give, however, Professor Schenck his due, he is reported to state that the publication of his asserted discovery is premature and was not made by his wish. Until, therefore, the sealed packet which he has handed to the Vienna Imperial Academy of Science is read in full, it will be best to reserve judgment. A Boston physician now claims that he has known how to produce boys at will for the past twenty-five years, but that he had refused to publish it for fear of the great harm it would do. Another American physician, a Dr. Wilbur Watson, of Danbury, Conn., has no such scruples, and boldly proclaims that he is able to control the sex of children. Unlike Professor Schenck, who it seems is in the main in accord with the generally received opinion of the influence of nutrition in the determination of sex, Dr. Watson holds that nutrition has nothing whatever to do with the matter, but refrains from making public his important secret. Unless he has substantial and sufficient reasons for withholding his knowledge, his action in so doing is ill-advised. If, as he asserts, he is absolutely convinced of his power to control sex, it is hard to understand why he should not be willing to reveal his discovery, and if he has not the courage of his convictions, he had no right to allow himself to be advertised as possessing the knowledge. In regard to the determination of sex, it is estimated that at the beginning of this century there were about 300 theories, and doubtless since that time the number has largely increased. Geddes and Thomson (in "Evolution of Sex") say that "throughout nature the influence of food is undoubtedly one of the most important environmental factors." According to Thury, an ovum fertilized soon after liberation tends to produce a female, while an older ovum will rather develop into a male. As a practical breeder, Thury claimed to determine sex of cattle upon this principle. Hoacker and Sadler were in favor of the generalization that when the male parent is the older the offspring are preponderatingly male, while if the parents be of the same age, or if the male parent be the younger, female offspring appear in increasing majority. The experiments of Henson (which supplement and extend those of Thury) show that a very favorable condition of both ovum and sperum will tend to the formation of a female. To again quote Thomson and Geddes, "The best known and probably still most influential theory is that of comparative vigor statistics, which seem to show that after an epidemic or a war the male births are in a greater majority than is usually the case, and 'vice versa.'" The influence of maternal nutrition as a most potent factor has been shown by repeated biologic experiments. Scientists, until most convincing proofs have been given, will remain sceptical as to the power of any person to control sex, and will regard with doubt assertions to that effect.—*Pediatics.*

